# Metabolic heterogeneity and cross-feeding within isogenic yeast populations captured by DILAC

**DOI:** 10.1038/s41564-022-01304-8

**Published:** 2023-02-16

**Authors:** Stephan Kamrad, Clara Correia-Melo, Lukasz Szyrwiel, Simran Kaur Aulakh, Jürg Bähler, Vadim Demichev, Michael Mülleder, Markus Ralser

**Affiliations:** 1 Department of Biochemistry, Charité Universitätsmedizin Berlin, Berlin, Germany; 2 Molecular Biology of Metabolism Laboratory, The Francis Crick Institute, London, UK; 3 Institute of Healthy Ageing and Department of Genetics, Evolution and Environment, University College London, London, UK; 4 Core Facility–High-Throughput Mass Spectrometry, Charité Universitätsmedizin Berlin, Berlin, Germany; 5 The Wellcome Centre for Human Genetics, Nuffield Department of Medicine, University of Oxford, Oxford, UK; 6 Max Planck Institute for Molecular Genetics, Berlin, Germany

**Keywords:** Microbial communities, Proteomics, Metabolomics, Metabolism

## Abstract

Genetically identical cells are known to differ in many physiological parameters such as growth rate and drug tolerance. Metabolic specialization is believed to be a cause of such phenotypic heterogeneity, but detection of metabolically divergent subpopulations remains technically challenging. We developed a proteomics-based technology, termed differential isotope labelling by amino acids (DILAC), that can detect producer and consumer subpopulations of a particular amino acid within an isogenic cell population by monitoring peptides with multiple occurrences of the amino acid. We reveal that young, morphologically undifferentiated yeast colonies contain subpopulations of lysine producers and consumers that emerge due to nutrient gradients. Deconvoluting their proteomes using DILAC, we find evidence for in situ cross-feeding where rapidly growing cells ferment and provide the more slowly growing, respiring cells with ethanol. Finally, by combining DILAC with fluorescence-activated cell sorting, we show that the metabolic subpopulations diverge phenotypically, as exemplified by a different tolerance to the antifungal drug amphotericin B. Overall, DILAC captures previously unnoticed metabolic heterogeneity and provides experimental evidence for the role of metabolic specialization and cross-feeding interactions as a source of phenotypic heterogeneity in isogenic cell populations.

## Abstract (web summary)

Stable isotope-labelled amino acid incorporation into proteins reveals that genetically homogeneous yeast colonies contain metabolically distinct subpopulations that cross-feed each other and are phenotypically diverse.

## Main

Recent advances in single-cell biology increasingly shed light on heterogeneity among isogenic cells. For instance, individual cells heterogeneously express metabolic enzymes and stress-response genes, possibly indicating metabolic specialization and bet-hedging strategies^1–4^. Heterogeneity does in fact appear to be pervasive and is emerging as a cellular modulator of phenotypes at the population level^5–8^. Moreover, heterogeneity at the single-cell level is associated with medically relevant antimicrobial tolerance and resistance phenotypes^9,10^. Despite these advances, we lack a comprehensive understanding of the biological sources of heterogeneity. While it may have stochastic components, heterogeneity is also a selected property that promotes drug tolerance and can be advantageous for survival in stress situations^11–15^ because yeast cells isolated from more challenging environments show a higher degree of heterogeneity than those isolated from more constant environments^16,17^.

An important metabolic property that can cause single-cell heterogeneity is the metabolic specialization of cells caused by exchange of metabolites. Both prokaryotic and eukaryotic cells export a wide range of metabolites and can dynamically switch between self-synthesis and uptake of metabolites which, in turn, triggers wide-ranging physiological changes, alters gene expression on a genome-wide scale and affects stress and drug tolerance^7,18–24^. Work with synthetic yeast communities has revealed the substantial potential of cells to engage in metabolite exchange interactions specifically involving amino acids^25,26^. However, metabolic heterogeneity and metabolite exchange within isogenic subpopulations remain elusive and their investigation remains challenging in wild-type cells^27^. By exploiting differential incorporation of stable isotope-labelled amino acids into protein, we were able to uncover extensive metabolic and phenotypic heterogeneity in undifferentiated, wild-type yeast colonies. The potential for the formation of diffusion gradients in media and within colonies makes these promising models with which to study heterogeneity. We find evidence for heterogeneous amino acid utilization and ethanol cross-feeding, and show that this differentially affects proteome, physiology and drug tolerance.

## Results

### A proteomics method for detection of metabolic subpopulations

To investigate the heterogeneous amino acid biosynthetic metabolism we relied on the well-characterized metabolism of *Saccharomyces cerevisiae*, focusing on lysine. Specifically we exploited the situation that, despite being a lysine prototroph, *S. cerevisiae* takes up this amino acid and incorporates it into proteins when present in medium^28,29^. Indeed, lysine accumulates to higher levels intracellularly in consumers than in producers, with important consequences for stress resistance (‘lysine harvesting’^18^). Moreover, in *S. cerevisiae* laboratory strains lysine is not metabolized as a nitrogen/carbon source^30^, which enables the use of heavy isotopes without label leakage into wider carbon metabolism.

**Fig. 1:**
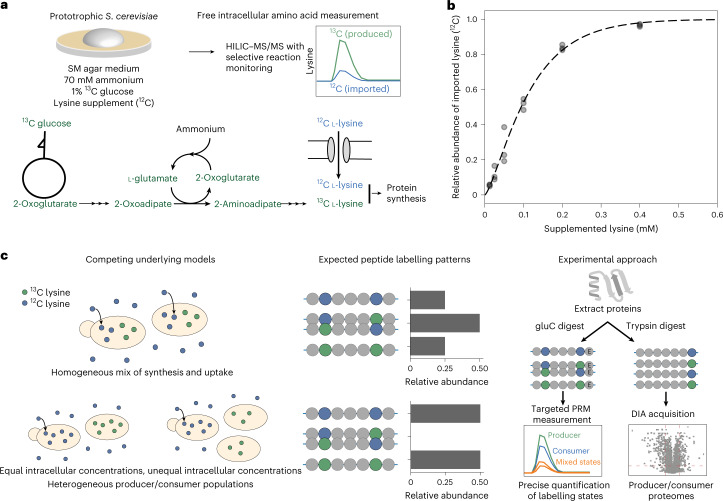
A strategy for the detection and analysis of metabolic subpopulations based on stable isotope incorporation and proteomics. **a**, Overview of model system. Yeast populations are grown on minimal media containing glucose and ammonium, with additional amino acid supplements (lysine in our example). Prototrophic yeast grown on these media therefore have two alternative routes of obtaining amino acids: (1) synthesis from glucose and ammonium using their own biosynthetic pathway (in the case of lysine, via the α-aminoadipate pathway) or (2) direct import of extracellular amino acids. The use of ^13^C-labelled glucose allows differentiation between amino acids obtained from these two routes via LC–MS/MS analysis of free intracellular amino acids. **b**, With increasing supplement concentration (*x* axis), yeast colonies progressively use amino acid import rather than synthesis (*n* = 3 biological replicates; a cumulative gamma distribution was fitted to data for visualization). **c**, Peptides carry signatures of metabolic subpopulations. Measurement of the population-averaged labelling state of free amino acids cannot determine the metabolic mode adopted by putative subpopulations. A population-level fraction of imported lysine of 0.5 (achieved with approximately 100 µM lysine supplementation (**b**)) could be explained by either (1) each cell importing half of its required lysine and synthesizing the other half or (2) distinct producer and consumer subpopulations (left). However, these two different underlying mechanisms can be distinguished at the peptide level when peptides with two or more lysine residues are considered. In the case of distinct producer/consumer populations, one would not expect to observe peptides with a mix of labelled and unlabelled lysine residues (middle). Here we employed two complementary experimental approaches to characterize lysine producer/consumer subpopulations: Protein extracts of yeast populations were digested with protease gluC, which selectively cuts after glutamate residues yielding a peptide mix, some of which contained two lysine residues that were then analysed with a targeted LC–MS/MS assay. Complementarily, protein extracts were digested with trypsin, which selectively cuts after lysine and arginine residues, resulting in a large number of peptides with a lysine residue at the C terminus that were then analysed using a data-independent acquisition scheme (DIA–PASEF^35^) and the software DIA–NN^32^.

**Extended Data Fig. 1:**
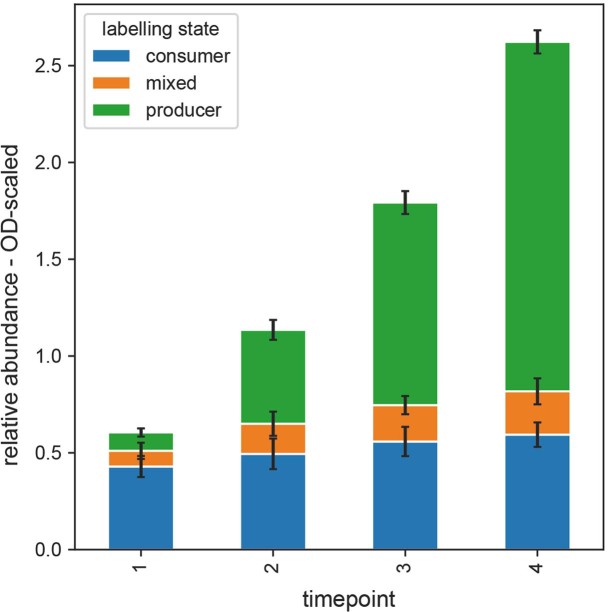
Producer/consumer dynamics in liquid batch cultures. Targeted measurements of labelling states in peptides (Methods, introduced later in the main text) were applied to liquid batch cultures grown in SM media with 1% ¹³C glucose and 68 μM unlabelled lysine. Bar heights indicate the average relative abundance of peptides originating from lysine producer and consumer cells. The data shown are the average across the top three fragments for three measured peptides and four biological replicates. Error bars indicate standard deviation. The relative abundances have been scaled to the OD of the culture at the time the sample was taken (that is the first sample was taken at an OD of approximately 0.6 and the final sample at approximately 2.5). The data shown indicate that initially almost all cells in the culture are lysine consumers. Then, a switch from uptake to synthesis occurs, indicated by the absolute abundance of consumer peptides not increasing after timepoint 1. Presumably, the lysine supplement has run out around the time the first sample has been taken and there is a net addition of only producer peptides.

We started by characterizing the synthesis-to-uptake switch in response to externally available lysine. We found that in liquid medium, supplemented lysine was rapidly consumed by all cells until exhausted, at which point cells turned from being consumers to producers (Extended Data Fig. 1). Subsequently, we focused on yeast colonies as a model for naturally spatially structured growth. Yeast colonies were grown on synthetic minimal (SM) medium with 1% glucose as the sole carbon source and ammonium as nitrogen source, as well as different lysine concentrations (Fig. 1a). To distinguish lysine molecules obtained by endogenous synthesis versus uptake, we employed a ^13^C-labelling strategy where cells were fed fully labelled ^13^C glucose and ^12^C lysine. We chose to use labelled glucose rather than labelled lysine for economic reasons, and because it allows easy application of the workflow to any amino acid. Lysine producer cells synthesize lysine from glucose and will hence contain lysine with heavy carbons (^13^C), while lysine consumer cells take up lysine directly from the medium and contain light (^12^C) lysine. Whole colonies were collected, free intracellular amino acids extracted and the population-wide ratio of labelled:unlabelled lysine was determined using liquid chromatography tandem mass spectrometry (LC–MS/MS)^31^. With increasing supplement concentrations, a larger fraction of the intracellular lysine is obtained by import rather than synthesis (Supplementary Dataset 1 and Fig. 1b; see Extended Data Table 1 for an overview of datasets generated in this study). Supplement concentrations in the micromolar range were sufficient to largely suppress synthesis and result in cells obtaining lysine essentially only by import, while a lysine concentration of approximately 100 µM resulted in half of the intracellular lysine in the population being obtained by import and the other by self-synthesis.

The latter could have resulted from two different scenarios where either (1) cells behave homogeneously, with all obtaining some lysine by import and some by synthesis or (2) subpopulations emerge, with some cells synthesizing lysine and others consuming it (Fig. 1c). To distinguish between the two scenarios we exploited the incorporation of labelled lysine into proteins and devised a method relying on peptides containing exactly two lysine residues. In a homogeneous population, cells contain a mix of produced and imported lysine molecules and the number of labelled lysine residues in a peptide should follow binomial distribution: in other words, in the case of a 1:1 ratio of synthesis and uptake, peptides with one out of two lysine residues labelled are expected to be twice as abundant as fully labelled or unlabelled peptides. If there are distinct producer and consumer subpopulations, these mixed-labelling states should be depleted and peptides should be either fully labelled or unlabelled (Fig. 1c).

### Young colonies contain producer and consumer subpopulations

**Fig. 2:**
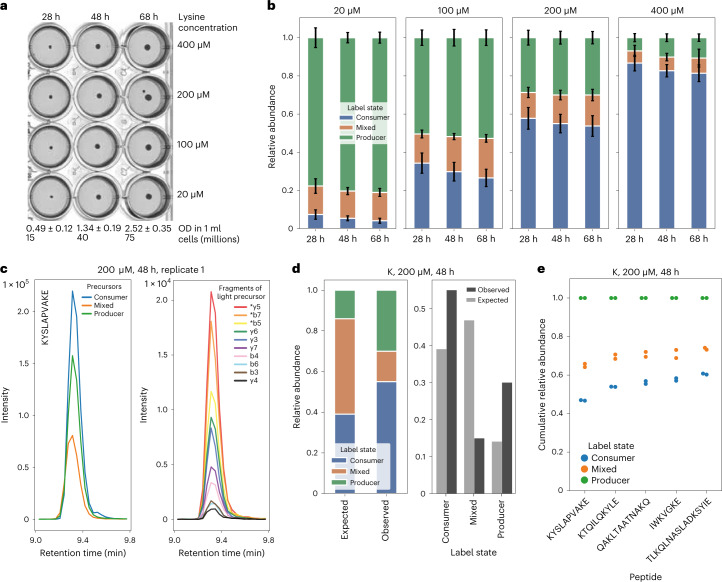
Yeast colonies contain stable lysine producer and consumer subpopulations. **a**, Proteomes of yeast colonies supplemented with four different lysine concentrations were collected at three different time points (*n* = 2 biological replicates). Colony population size increased throughout the experiment (measured by OD, with approximate conversion to cell numbers). Uncropped images are shown in Extended Data Fig. 2d. **b**, Labelling states of five peptides, each containing two lysine residues, were determined by targeted LC–MS/MS measurements. Shown is the relative abundance of producer, consumer and mixed peptides over time and with varying lysine supplement concentration. Ratios are largely stable over the three time points and depend primarily on lysine supplement concentration in the medium. Bar heights show the mean across the top three fragments and across five measured peptides and two biological replicates; error bars indicate s.d. **c**, Chromatograms (representative examples) illustrating how peptide ratios were determined analytically. Left, chromatograms (ion intensity over time) for +2 charged precursors with modification-stripped sequence KYSLAPVAKE (single letter amino acid code). Producer, consumer and mixed peptides are distinguishable by their mass:charge ratio. To confidently identify peptides and resolve the two mixed-labelling states, precursors were fragmented in the mass spectrometer. Right, fragment chromatograms for the consumer peptide. The top three most abundant fragments from those containing exactly one lysine were used for quantification (marked with an asterisk). **d**, Mixed-labelling-state peptides are substantially under-represented, indicating the existence of distinct producer and consumer subpopulations. The ‘expected’ distribution of labelling states, under the assumption of metabolic homogeneity, was calculated as a binomial distribution with a probability value taken from the overall fraction of labelled lysine sites. The 48 h timepoint of colonies supplemented with 200 µM lysine is shown as an example. **e**, Variation between measured peptides is greater than that between biological replicates. Shown are cumulative relative abundance fractions of the three labelling states for each replicate and peptide. From left to right, peptides show increasing fractions of consumers, which may indicate that the proteins from which these peptides originate are differentially expressed in producers and consumers.

To investigate whether yeast colonies contain lysine producer and consumer subpopulations, we used SM agar medium with isotope-labelled glucose (^13^C) supplemented with four different lysine (^12^C) concentrations ranging from 20 to 400 µM and grew colonies for 28, 48 and 68 h. Throughout this time course, growth of colonies took place (Fig. 2a). Proteins of whole colonies were then extracted and digested into peptides using protease gluC and subjected to targeted LC–MS/MS analysis.

**Extended Data Fig. 2:**
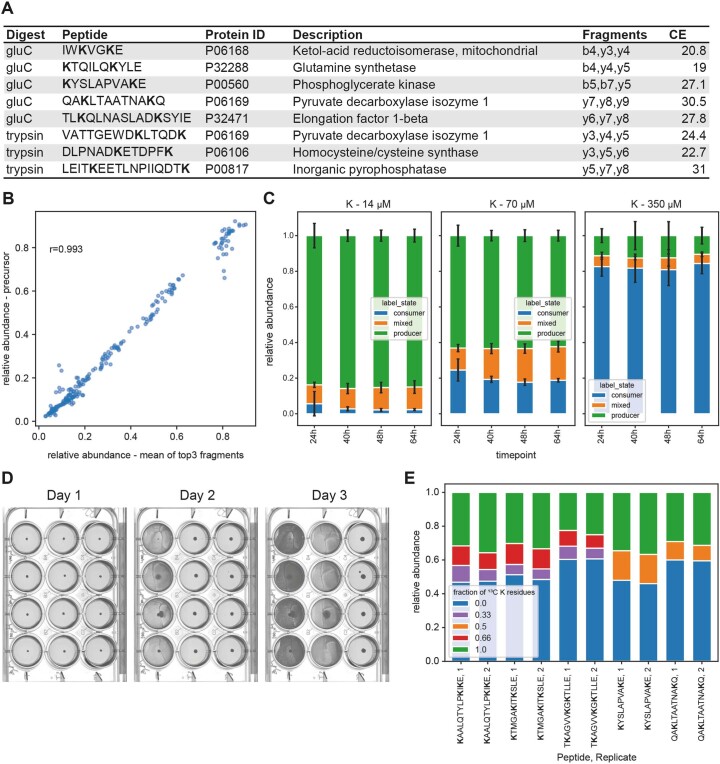
Targeted proteomics (Dataset 2). **a**, Table listing peptides used for quantifying producer, consumer and mixed populations by targeted proteomics (PRM) assays. The Fragments column lists the top3 fragments not shared by the two isobaric mixed labelling state precursors, which were used for quantification. The CE column indicates the collision energies used, as predicted by Skyline. **b**, Similar results are obtained with quantification of intact precursors as with the top3 suitable fragments. Shown are all individual data points from Dataset 2. The Pearson correlation between both quantification strategies is 0.993. Quantification at MS1 level can be useful when the signal is low, as was the case for FAC-sorted populations shown in Fig. 4. **c**, Similar results are obtained when peptides with missed cleavages from tryptic digests are used. The data shown is from an independent experiment otherwise similar to the one shown in Fig. 2. The data shown is the average of the top three fragments of three measured peptides across two biological replicates. Error bars show the standard deviation. **d**, Uncropped images of colonies grown in a 12-well plate (cropped version shown in Fig. 2A). Each day, the plate was scanned and a column of colonies was harvested (right to left), resulting in a pseudo timecourse. **e**, Targeted proteomics experiment investigating peptides with three lysine residues. Two biological replicate samples (200 μM lysine, 48 h) from the experiment shown in Fig. 2 were remeasured with a targeted method that included peptides with three lysine residues (as well as two peptides with two lysine residues, as control). MS1 data was used for quantification. The depletion of the mixed labelling states is apparent also in peptides with three lysine residues.

We determined the relative abundance of producer, consumer and mixed-labelling states of five abundant and reliably measured peptides with exactly two lysine residues each (Methods, Supplementary Dataset 2 and Extended Data Fig. 2a). We illustrate these states averaged across the top three suitable fragments, the five peptides and two biological replicates (Fig. 2b), as well as example data obtained from the LC–MS/MS experiment (Fig. 2c). With rising lysine concentration an increasing fraction of lysine residues was light (^12^C), which indicates cells obtaining lysine by import rather than synthesis (concordant with Fig. 1b). The ratio of consumer, producer and mixed peptides was approximately stable over the three timepoints. Strikingly, the mixed-labelling state, expected if cells synthesize part of their lysine and uptake another part, was rare compared with uniform-labelling states. This can be quantified by comparison with expected relative abundances under the homogenous model: for colonies supplemented with 200 µM lysine and harvested after 48 h, the overall fraction of imported (^12^C) residues across all labelling states was 62.5%. From this, one can compute an expected abundance of 46.9% for mixed-labelling states using a simple Bernoulli model (Methods). This is approximately three times higher than the observed abundance of 15.0%, indicating that a homogeneous model cannot account for these data (Fig. 2d); in other words, our data indicate that the individual cell either produces or consumes lysine and that mixed states are not abundant.

**Extended Data Fig. 3:**
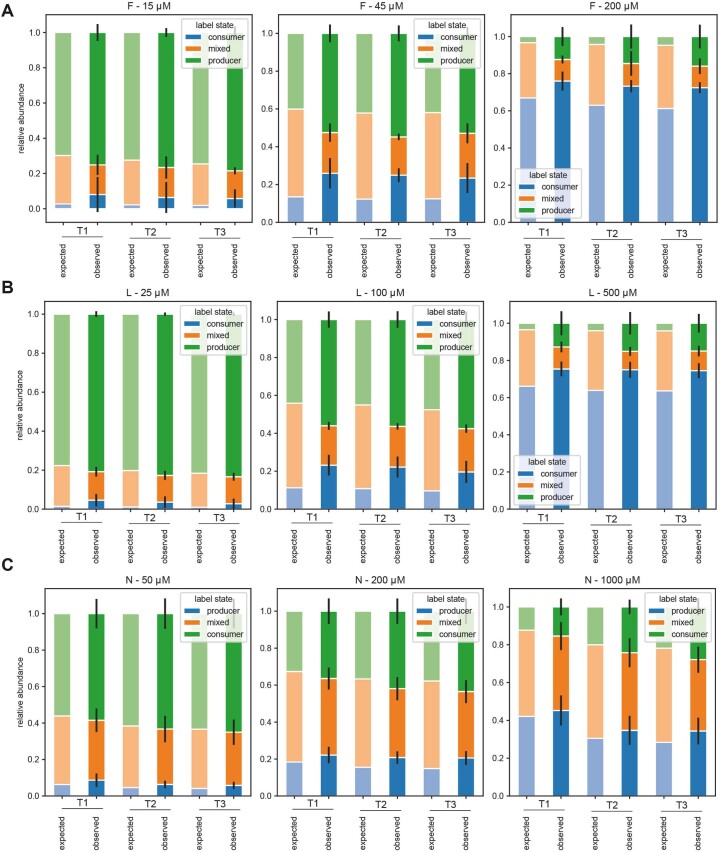
Producer and consumer subpopulations also emerge with other amino acid supplements. **a**–**c**, Targeted proteomics was used to determine the relative abundance of labelling states in colonies supplemented with three different amino acids (F - phenylalanine (panel **a**); L - leucine (panel **b**); N - asparagine (panel **c**)). For each supplementation regime, a colony (n = 1 biological replicate) was harvested on three consecutive days (T1, T2, T3) and proteome samples prepared by tryptic digestion. MS2-level relative abundances were averaged across the top3 fragments of at least four peptides. Bar heights indicate the mean across fragments of all peptides, errorbars indicate the standard deviation. All underlying data is available through Panorama Public.

To rule out experimental artefacts we determined labelling states using three alternative approaches. First, similar labelling ratios were quantified from intact precursors without resolving the two mixed-labelling states (Extended Data Fig. 2b). Second, we quantified labelling states from tryptic digests using missed cleavage peptides, removing the need for the less common protein digestion with protease gluC (Extended Data Fig. 2c). Third, congruent results were obtained when peptides with three lysine residues were investigated (Extended Data Fig. 2e). We additionally conducted similar experiments with three other amino acids and observed producer/consumer subpopulations for two of these (leucine and phenylalanine, but not asparagine; Extended Data Fig. 3). Hence, our data paint a cohesive picture where subpopulations of cells within colonies either consume or produce lysine, leucine or phenylalanine.

We noted a relatively high variation in the labelling data (expressed as error bars in Fig. 2b) and set out to determine its source. Plotting peptides and replicates individually for the 200 µM supplementation concentration, we noted that the replicates agree very closely and that the variation noted emerges at the peptide level (Fig. 2e). Since most of these peptides belong to metabolic enzymes (Extended Data Fig. 2a), this raised the interesting hypothesis that these proteins might be expressed to different levels in producers and consumer cells, and that proteomic technology could characterize the differentially labelled proteomes of these subpopulations.

### Proteome-wide labelling reveals subpopulation gene expression

**Extended Data Fig. 4:**
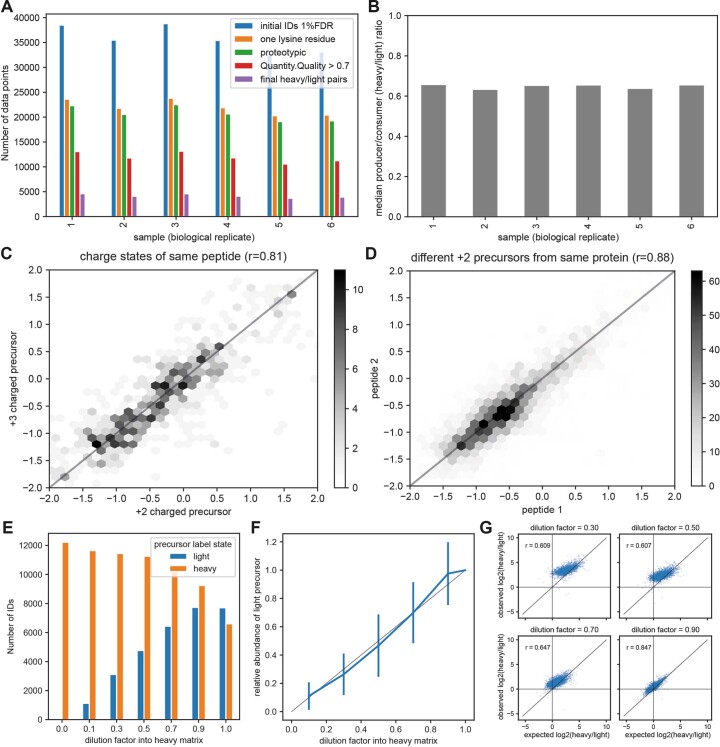
Proteome-wide deconvolution of producer and consumer proteomes by DIA-PASEF (Dataset 3). **a**, Number of precursor IDs across samples. The progressive filters which were applied are shown in different colours. **b**, The median ratio of producer to consumer (heavy to light) peptides was highly consistent across biological replicates. **c**, After suitable quality filters were applied (in particular: Quantity.Quality > 0.7), log2-transformed labelling ratios between +2 and +3 charge states of the same peptide correlated tightly (Pearson r = 0.81). (D) Similarly, log2-transformed labelling ratios of different proteotypic, tryptic peptides from the same protein correlated tightly (Pearson r = 0.88). This analysis was restricted to +2 charged precursors, identified across all samples. In cases where more than two precursors were found for a protein, an arbitrary pair was selected. **e**, To further test the robustness of the analytical approach, a pooled sample was mixed with fully ¹³C-labelled peptides at different ratios. Identical quality filters were applied as before. The number of light precursors (¹²C-lysine) identified decreases with dilution and virtually no light peptides are wrongly identified in the fully labelled matrix. **f**, Detected abundances of light peptides (relative to abundance in undiluted sample) behave proportionally to the dilution factor. Error bars show median absolute deviation across all peptides identified in the sample. **g**, The original labelling ratios can be recovered with reasonable accuracy (the Pearson correlation coefficient is shown) even upon three-fold dilution into heavy matrix although at low dilutions there is a tendency to overestimate ratios and the observed ratios show a lower degree of variance (compression of ratios) compared to what is expected.

**Extended Data Fig. 5:**
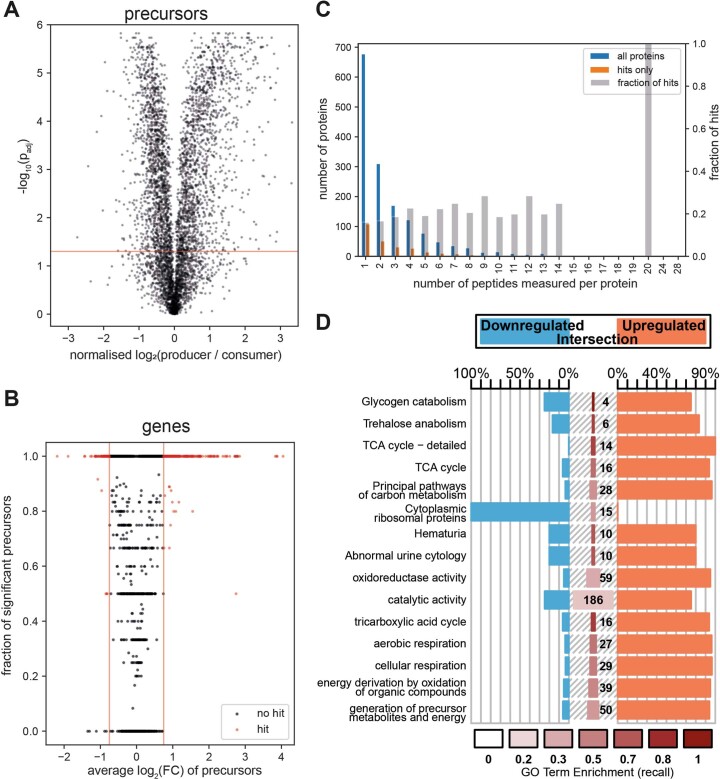
Hit calling and GO enrichment (Dataset 3 continued). **a**, Volcano plot illustrating hit calling at the peptide level (one-sample, two-sided t-test, corrected for multiple testing by Benjamini-Hochberg method, further described in Methods). **b**, Volcano plot illustrating hit calling at the gene level, as described in the main text. **c**, Barplot illustrating the number of measured precursor pairs per protein for all proteins in the dataset (blue) and only the proteins found to be differentially expressed (orange). The hit frequency (grey) is approximately constant, indicating that proteins with fewer identified peptides (less reliably measured) are not more likely to be a hit. **d**, CellPlot illustrating GO enrichment analysis of all genes differentially expressed in producers and consumers. GO enrichment was performed in gProfiler, using all measured proteins as the background. Only significant (p_adj_ < 0.05, using the g_SCS method of gProfiler) terms are shown. Recall refers to the fraction of genes annotated to the term and present in the background which were a hit in Dataset 2, the intersection is their absolute number.

Differential labelling of the proteomes of lysine consumers and producers opened the possibility of deconvoluting their distinct proteomes from bulk measurements without the need to separate cells. We therefore expanded our analytical approach to capture thousands of peptides using parallel accumulation–serial fragmentation combined with data-independent acquisition (DIA–PASEF), followed by data-independent–neural network (DIA–NN) analysis^32–35^ (Fig. 1c). We grew colonies on agar with ^13^C glucose and 200 µM lysine and acquired deep proteome profiles for six colonies grown for 48 h. In total, we detected approximately 35,000 precursors per sample at 1% false discovery rate, containing 3,600–4,600 high-quality, proteotypic light/heavy pairs containing exactly one lysine residue (Supplementary Dataset 3, Methods and Extended Data Fig. 4a). The median ratio of producer- to consumer-attributable peptides ranged between 0.63 and 0.66 across the six samples (Extended Data Fig. 4b). This is consistent with the observation that consumers are slightly more abundant at this supplement concentration in colonies (Fig. 2). The quality of the data was confirmed by the correlation of the labelling state of peptides derived from the same protein, and of the same precursor at +2 and +3 charge states (Extended Data Fig. 4c,d), as well as by a spike-in experiment (Extended Data Fig. 4e–g). Out of 4,327 heavy/light precursor pairs identified in at least three replicates, 3,044 had labelling ratios significantly different from the median producer/consumer ratio (Extended Data Fig. 5a; false-discovery-rate-corrected, one-sample, two-sided *t*-test). A protein was considered differentially expressed between producers and consumers if (1) at least one of its precursors was significant in the previous analysis, (2) all precursors showed the same trend and (3) the average absolute log_2_-transformed fold change (FC) across precursors was >0.75 (Extended Data Fig. 5b). Due to this stringent filtering we obtained summary statistics for 1,546 proteins, of which 277 were significantly differently expressed between producers and consumers. There was no obvious relationship between the number of peptides measured per protein and the likelihood of the protein being a hit, indicating that less reliably measured proteins with few measured peptides are not over-represented in the results (Extended Data Fig. 5c).

**Fig. 3:**
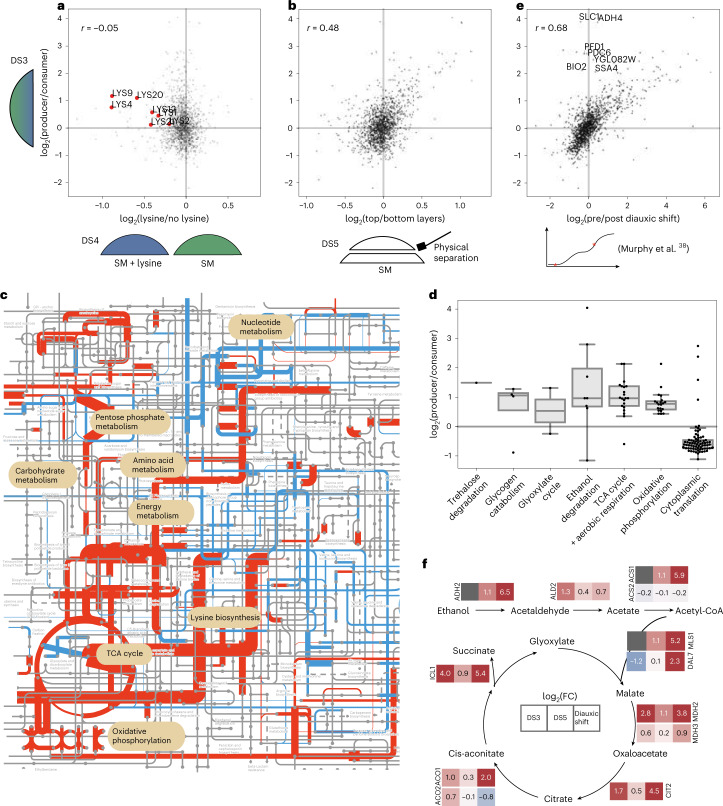
Differential proteome analysis of lysine producers and consumers reveals extensive diauxie-like heterogeneity in young colonies. **a**, The lysine biosynthesis pathway is upregulated in producer cells, but their overall proteome profile is not explained by lysine producer/consumer status. The *y* axis shows protein abundance ratios in producer versus consumer cells within colonies supplemented with 200 µM lysine, determined from the ratio of lysine heavy versus light peptides by DILAC (Supplementary Dataset 3 (DS3); *n* = 6 biological replicates). The *x* axis shows abundance ratios from a separate experiment comparing colonies grown on unlabelled medium with and without 400 µM lysine supplement (Supplementary Dataset 4 (DS4); *n* = 5 biological replicates). While the lysine biosynthesis pathway is concordantly affected, the overall correlation is low, indicating that lysine availability alone is not the main driver of producer/consumer proteome differences. **b**, Producer/consumer proteome profiles correlate with proteomes from top and bottom layers of colonies. In an independent experiment (*x* axis, Supplementary Dataset 5 (DS5); *n* = 7 biological replicates), colonies were grown on unlabelled and non-supplemented medium; the top layer of cells was then removed using a cell scraper and both top and bottom layers analysed separately. **c**, Differences in producer/consumer proteomes (Supplementary Dataset 3) mapped to metabolic pathways using iPATH^80^ indicate strong and concordant changes in expression of key metabolic pathways. The colour of edges indicates the direction (red, up; blue, down) and edge width reflects the magnitude of change. **d**, Changes in expression of proteins of key metabolic pathways between producers and consumers (Supplementary Dataset 3). Following convention, box-plot elements are defined as follows: centre line, median; box limits, upper and lower quartiles; whiskers, 1.5× interquartile range; points, outliers. **e**, Producer/consumer protein ratio profiles (Supplementary Dataset 3, *y* axis) correlate with changes in gene expression associated with the diauxic shift observed in liquid cultures^38^. **a**,**b**,**e**, Pearson correlation coefficient is shown. **f**, Pathway map illustrating changes in expression of the ethanol degradation pathway and glyoxlyate cycle in three datasets (left to right): producer/consumer protein ratios obtained with DILAC (Supplementary Dataset 3), top and bottom layer in non-supplemented, unlabelled medium (Supplementary Dataset 5) and proteome changes from postdiauxic versus early exponential growth^38^.

We first tested whether these differences in gene expression could be directly attributed to the lysine production/consumption status of cells. For this we generated an independent dataset comparing whole-colony, unlabelled proteomes of colonies supplemented with and without 400 µM lysine (a concentration at which almost all cells are consumers) (Supplementary Dataset 4). Following comparison of this profile with the producer/consumer profile obtained by ^13^C labelling (Supplementary Dataset 4 versus 3), enzymes involved in lysine biosynthesis were found to be strongly upregulated in lysine producers in both datasets (Fig. 3a). This was expected because the lysine biosynthesis pathway is under transcriptional control of the transcription factor Lys14p^36^. However, beyond this no correlation was observed (global correlation *r* = −0.05, Pearson correlation). This result indicated that a broader range of factors beyond lysine consumer/producer state affect the proteome of cells growing within the colony.

### Isogenic, young colonies contain fermenting and respiring cells

The set of proteins differentially abundant in producer and consumer cells within the colony were enriched for genes annotated to central carbon and energy metabolism (Extended Data Fig. 5d). We speculated that other nutrient gradients could form within the colonies, driving this differentiation. If this were the case we would expect the consumers to be mainly located towards the bottom of the colony, close to the nutrient source where nutrient concentrations are probably highest. To directly test for this possibility, we independently obtained proteomes for cells in the top and bottom parts of seven colonies grown on standard (unlabelled and non-supplemented) media. A reproducible separation of cells was achieved using a custom-made plastic guide and a cell scraper (Methods and Fig. 3b). Proteins were extracted, tryptic peptides generated and proteomes measured separately for each layer of the colony (Supplementary Dataset 5). The differential expression profile of top and bottom cells correlated with that obtained previously for producer/consumer cells by ^13^C labelling (*r* = 0.48, Pearson correlation; Supplementary Dataset 5 versus 3, and Fig. 3b). This result confirmed that lysine producers are located predominantly in the top region of colonies and that, even in the absence of lysine supplementation, substantial proteomic heterogeneity exists within young colonies.

We next focused on functional differences in the proteomes of the subpopulations. Mapping of quantitative gene expression changes obtained by differential isotope labelling by amino acids (DILAC; Supplementary Dataset 3) on the metabolic network (Fig. 3c) revealed substantial and concordant abundance changes in enzymes participating in central carbon and energy metabolism. Notably, the tricarboxylic acid (TCA) cycle, oxidative phosphorylation and parts of the pentose phosphate pathway were upregulated while cytoplasmic translation was downregulated in lysine producer cells located in the upper layer (Fig. 3d). Lysine-consuming cells in the bottom layer, on the other hand, had a proteomic signature of rapidly growing, fermenting yeast. We and others have previously observed similar gene expression signatures in batch cultures where cells rely either on respiration or fermentation for growth and energy production^37–39^. Furthermore, there could be a direct influence of lysine, the lack of which requires increased proteome allocation to lysine synthesis at the expense of proteins involved in translation^40^. Overall, these results indicated that metabolic states that occur sequentially (temporally separated) in batch culture co-occur within colonies in a spatially separated manner.

**Extended Data Fig. 6:**
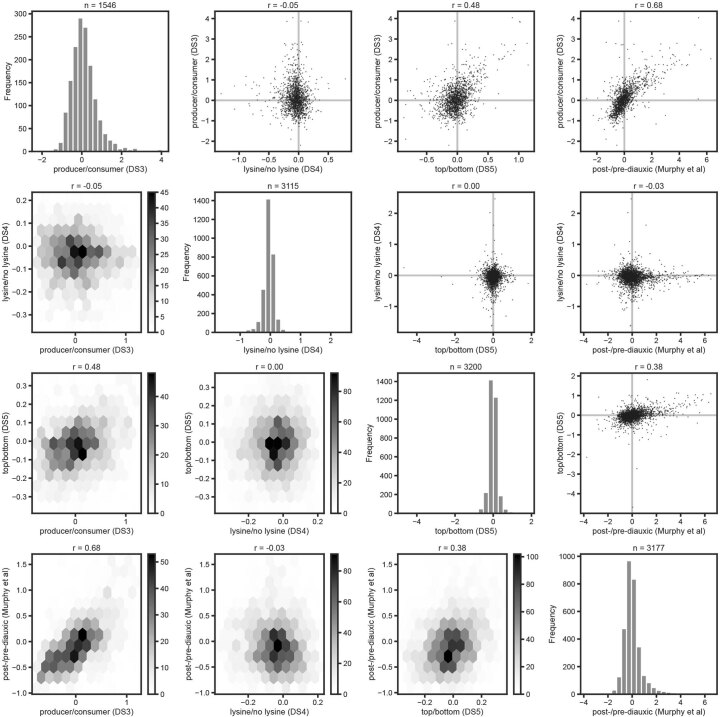
Comparative analysis of producer/consumer proteome signatures. Shown are pairwise correlation plots (as scatter plots and hexbin plots to visualise strong responders and the general trend respectively) for different datasets collected in this study, as well as a dataset that describes proteome response to diauxie (calculated from data published by Murphy et al. 2015). The Pearson correlation is indicated above each plot. All datasets consist of log_2_-transformed fold changes. The distribution and total number of data points is shown in histograms on the diagonal.

We therefore compared producer/consumer proteome profiles (obtained from actively growing colonies) with the proteomic changes observed between early exponential and postdiauxic growth (postfermentative growth on a non-preferred carbon source such as ethanol) in liquid batch culture (published dataset by Murphy et al.^38^). The global correlation of the two datasets was high (*r* = 0.68, Pearson correlation; Fig. 3e and Extended Data Fig. 6), with several of the key metabolic changes that differentiate exponential with postdiauxic shift cells represented in the lysine consumer versus producer profiles. For instance, in the pathway converting ethanol to acetyl coenzyme A (acetyl-CoA), elevated enzyme levels were detected for all three reactions (Fig. 3f): alcohol dehydrogenase Adh2p (Supplementary Dataset 3: not measured; Supplementary Dataset 5: FC = 2.14, *P*_adj_ = 0.0003); the acetaldehyde dehydrogenase Ald2p (Supplementary Dataset 3: FC = 2.48, two of two peptides were significant; Supplementary Dataset 5: FC = 1.37, *P*_adj_ = 0.002); as well as the acetyl-CoA synthetase Acs1p (Supplementary Dataset 3: not measured; Supplementary Dataset 5: FC = 2.08, *P*_adj_ = 0.0005), but not its isoform Acs2p. This is consistent with the shift from fermentation to oxidative metabolism, because Acs1p is known to be the glucose-responsive isoform while Acs2p is thought to be regulated in response to lipid metabolism^41^. Growth on two-carbon compounds (such as ethanol or acetate) requires that acetyl-CoA is further metabolized by the glyoxylate cycle^42^ which, contrary to the TCA cycle, does not include decarboxylation reactions and therefore allows the net generation of four-carbon from two-carbon compounds. The glyoxylate cycle is repressed in the presence of glucose^43^. We note a strong upregulation of glyoxylate cycle genes in both lysine producer cells (Supplementary Dataset 3) and top-layer cells (Supplementary Dataset 5 and Fig. 3f).

Thus, both on their own and in comparison with publicly available data, our results paint a consistent picture: even within young, morphologically undifferentiated colonies there is metabolic compartmentalization. Cells close to the agar surface have preferential access to nutrients, including glucose and amino acids, and grow by fermentation while cells in the upper layer respire and are required to produce lysine. Metabolic processes that have long been described as occurring in a temporally separated manner in liquid batch cultures appear to happen simultaneously but spatially separated within the colony. This is further supported by the recent discovery of ethanol as a shared resource in *S. cerevisiae* cultures^44^, and is analogous to acetate cross-feeding in *Escherichia coli* colonies^45–47^. Of note, this metabolic specialization is established long before the emergence of morphological differentiation of ageing yeast colonies^48^.

### Ion and vitamin gradients add to physiological diversity

The overall proteome profiles between lysine producers/consumers and exponential phase/postdiauxic cells were correlated. However, we observed other striking differences in colony metabolism not reflected in batch culture experiments and not explained by lysine availability (Supplementary Note 1). Out of seven proteins strongly differentially expressed within colonies (Supplementary Dataset 3; abs(log_2_(FC)) > 1.5) but not between pre- and postdiauxic cells (abs(log_2_(FC)) < 0.5 (ref. ^38^); Fig. 3f), three stood out because they showed concordant changes in Supplementary Dataset 5 (physically separated top/bottom cell layers). Most notably, Adh4p, a minor alcohol dehydrogenase isozyme, is upregulated in lysine producer cells (Supplementary Dataset 3; FC = 14.6, four of four peptides significant). Adh4p is known to be induced by zinc starvation^49^, which could indicate that top cells are zinc starved. Bio2p, a key biotin biosynthetic enzyme, was also strongly upregulated in producer cells (Supplementary Dataset 3; FC = 4.3, one of one peptide significant), which could indicate that top cells have reduced access to biotin as compared with bottom cells. Furthermore, upregulation of the chaperone Ssa4p (Supplementary Dataset 3; FC = 4.2, five of five peptides significant) suggests that top cells face stresses that are different from postdiauxic cells in liquid cultures. Further experiments will be required to confirm the presence, and explore the consequence, of additional nutrient gradients in colonies.

### Intracolony heterogeneity alters resistance to amphotericin B

**Fig. 4:**
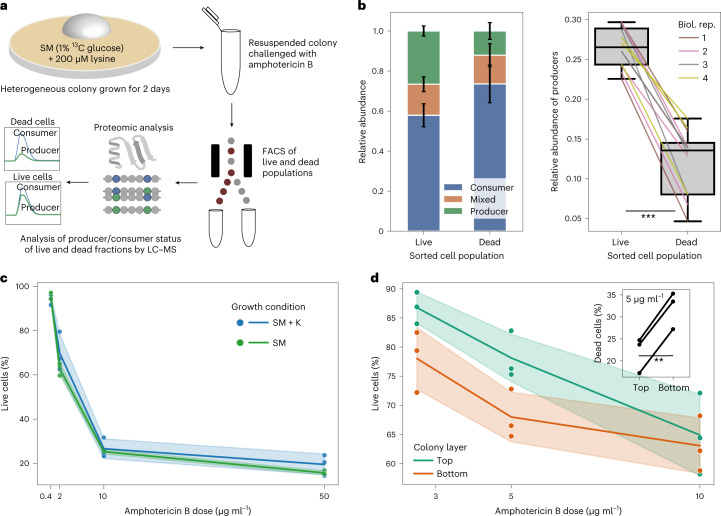
FACS and proteomics link producer/consumer status to an antimicrobial resistance phenotype. **a**, Experimental scheme used to test for phenotypic differences between producer and consumer subpopulations. A ^13^C-labelled, heterogeneous population of cells from a colony is resuspended and challenged with the fungicidal drug amphotericin B. Live and dead populations are then separated physically using FACS and their producer/consumer status determined using targeted proteomics as before. This method can directly link a metabolic phenotype to one with a fluorescent readout. **b**, Lysine producer cells of colonies were found to be significantly more likely to survive amphotericin B treatment (mean fraction of producer cells in live population 26.6% versus 12.1% in the dead population; *P* = 0.0004, paired two-sided *t*-test, *n* = 4 biological replicates (biol. rep.)). Error bars indicate s.d. Lines indicate peptide pairs across colour-coded biological replicates. Following convention, box-plot elements are defined as follows: centre line, median; box limits, upper and lower quartiles; whiskers, 1.5× interquartile range; points, outliers. **c**, This difference in susceptibility is not due to lysine production alone. No clear difference in susceptibility was observed when whole colonies, which were either producers (no supplement) or consumers (400 μM lysine), were challenged with varying concentrations of amphotericin B. **d**, Difference in susceptibility is linked to the position of cells in the colony. Physically separated top and bottom cells from non-supplemented colonies were challenged with various concentrations of amphotericin B. Bottom-layer cells were significantly more susceptible (at 5 μg ml^–1^: mean fraction of dead cells in bottom layer 32% versus 22% in the top layer; *P* = 0.0005, paired two-sided *t*-test (see inset)). **c**,**d**, Lines and shaded areas represent mean and s.d., respectively. **P < 0.005, ***P < 0.0005.

**Extended Data Fig. 7:**
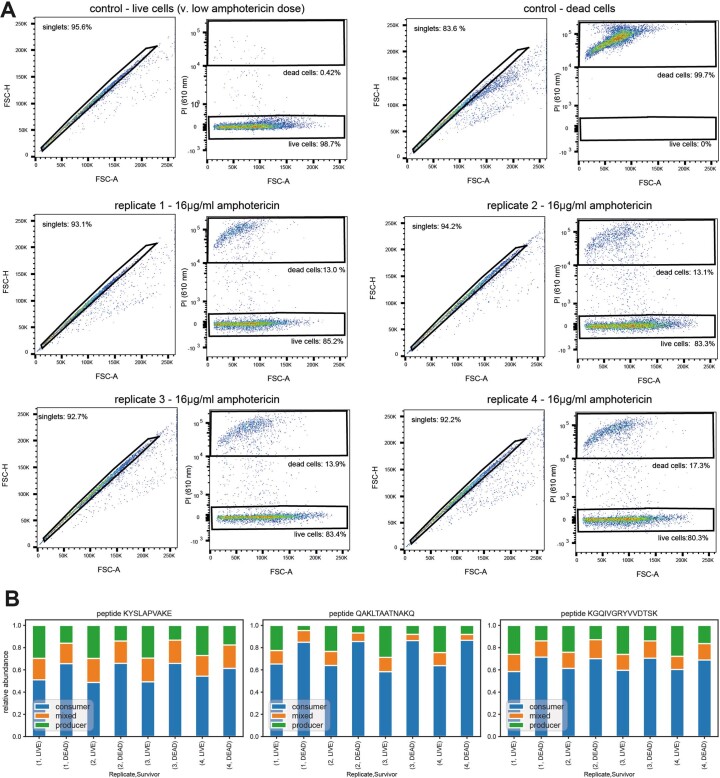
Targeted proteomic analysis of producer/consumer status of live/dead sorted populations after amphotericin B treatment. **a**, Plots illustrating gating strategy for FACS of cells originating from heterogeneous ¹³C-labelled colonies challenged with amphotericin B and stained with propidium iodide (PI). 10,000 events were recorded to check viability and gating of each sample, after which a minimum of 4 million cells were sorted per population. Singlets were gated based on FSC-A (forward scattered channel - area) and FSC-H (forward scattered channel - height) intensities. Conservative live and dead population gates were defined based on the FSC-A and 610 nm channels. **b**, Quantification of producer and consumer peptides in live and dead populations obtained by FAC-sorting. Each plot shows the data for a single peptide, quantified at the precursor level using a targeted proteomics (PRM) workflow. Dead cell populations consistently contain fewer peptides originating from producer cells.

**Extended Data Fig. 8:**
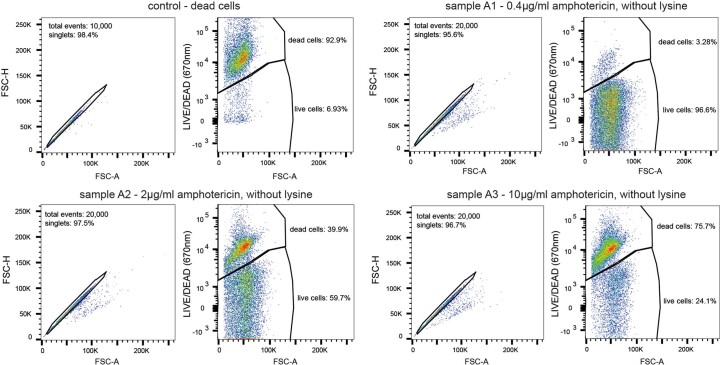
Flow cytometric cell viability analysis of resuspended colonies grown with and without lysine after challenge with amphotericin B. 20,000 events were recorded per sample (fewer for the controls). Singlets were gated based on FSC-A (forward scattered channel - area) and FSC-H (forward scattered channel - height) intensities. The median singlet rate across the 24 samples was 95.8% with a minimum of 84.4%. Live and dead population gates were defined based on the live and dead controls using FSC-A and 670 nm channels. Identical gates were applied to all samples. Three samples are shown as examples.

**Extended Data Fig. 9:**
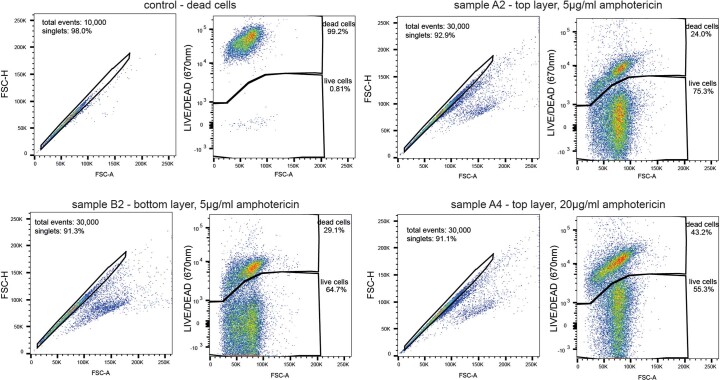
Flow cytometric cell viability analysis of resuspended top and bottom layers of colonies challenged with amphotericin B. Thirty thousand events were recorded per sample (fewer for the controls). Singlets were gated based on FSC-A (forward scattered channel - area) and FSC-H (forward scattered channel - height) intensities. The median singlet rate across the 24 samples was 92.8% with a minimum of 90.5%. Live and dead population gates were defined based on the live and dead controls using FSC-A and 670 nm channels. Identical gates were applied to all samples. Three samples are shown as examples.

We next wondered whether metabolic heterogeneity within colonies could affect cellular phenotypes (Fig. 4a). A homogenized (resuspended) heterogeneous population (obtained from a colony grown on medium with ^13^C glucose and 200 μM lysine supplement) was subjected to amphotericin B for one h and stained with the membrane permeability dye propidium iodine, which marks dead cells that have lost membrane integrity (Extended Data Fig. 7a). We then separated live and dead cells using fluorescence-activated cell sorting (FACS) and determined labelling states of both groups separately. Cells that did not survive drug treatment were significantly less likely to be producer cells (*P* = 0.0004, paired two-sided Student’s *t*-test; Fig. 4b and Extended Data Fig. 7b). We then investigated whether this was a direct consequence of lysine consumption, but found no apparent difference in susceptibility between colonies grown either in non-supplemented medium or in medium supplemented with 400 μM lysine (Fig. 4c and Extended Data Fig. 8). Rather, the difference in susceptibility is explained by the position of cells within the colony (Fig. 4d and Extended Data Fig. 9; physically separated top and bottom layers from non-supplemented colonies). These results demonstrate the ability of DILAC to link metabolic and resistance phenotypes at the subpopulation level, and add evidence linking metabolic state and drug resistance in isogenic populations.

## Discussion

Detection of metabolic heterogeneity and metabolic interactions within communities remains challenging. Even within multispecies communities, genomic information (for example, the presence or absence of a particular biosynthetic pathway) is not sufficient for obtaining a comprehensive picture of metabolic exchange interactions because auxotrophs and prototrophs alike import extracellular metabolites. As a consequence, we have only sparse evidence regarding the underlying mechanisms and physiological consequences of metabolic heterogeneity. Here we have developed DILAC, a method for the characterization of metabolic subpopulations. DILAC captures differences in amino acid consumption and production by quantifying the differential incorporation of stable isotope-labelled amino acids into peptides with multiple occurrences of the same amino acid. DILAC can hence discriminate, from proteomics data measured in bulk, whether a (sub)population of cells produced, consumed—or both consumed and produced—a particular amino acid. Indeed DILAC is complementary to previous approaches that combined ^13^C labelling and proteomics for metabolic flux analysis^50^ and investigations of substrate preferences^51–56^, via its unique ability to directly detect heterogeneous amino acid metabolism across isogenic and morphologically homogeneous populations of wild-type cells. Moreover, based on differential labelling patterns, DILAC can deconvolute the proteomes of producer and consumer cells out of bulk measurements and, hence, determine which proteins are differentially expressed between subpopulations without separating them physically.

The DILAC workflow is in principle broadly applicable but comes with certain technical and biological limitations. The detection of heterogeneity by targeted analysis of the depletion of the mixed-labelling state is qualitative in this study and might be probelematic in cases where subpopulations are not as distinct—that is, separated by gradual transitions. Furthermore, DILAC is not precise in the quantification of the number of cells in each subpopulation because these can have different protein synthesis rates and must therefore be complemented by other methods (for example, single-cell metabolomics^57^ and proteomics) if precise ratios between cell types are to be determined. Moreover, although application of DILAC to other amino acids has been demonstrated here, the depth and precision of proteome-wide profile deconvolution vary depending on the prevalence of suitable peptides and fragments. Furthermore, for technical reasons, the characterization of rare subpopulations will remain more challenging compared with high-abundance subpopulations because quantitative precision and the number of identified peptide pairs diminish for heavily skewed heavy/light ratios.

A high degree of metabolic diversity was revealed by DILAC within young, undifferentiated yeast colonies. It has long been known that growth of microbial colonies is limited by diffusion of metabolic substrates^58,59^, and this could mean that only a subpopulation of cells proliferates^60,61^. Our data conclusively describe subpopulations, stable in relative size, that produce and consume lysine and whose proteomes simultaneously carried a strong signature of fermentative growth on glucose and respiratory growth on ethanol. Colonies, arguably replicating aspects of spatial structure and chemical gradients naturally encountered by yeasts in the absence of artificial homogenization by rapid shaking, are more complex and metabolically heterogeneous than previously thought. This adds to related studies in bacteria, in which recent work has described the cross-feeding of alanine^62^ and acetate^45–47^. We also conducted experiments in liquid batch culture, and here our results help to explain why experimental results can differ markedly between different points in growth phases. We find that cells switch from a consumer to a producer state at low supplement concentration and, because different amino acid supplements in batch culture are consumed rapidly but at different rates^63^, different points in batch culture growth experiments will be composed of different consumer and producer populations. Furthermore, our results are relevant for microbiological experiments using colonies. These typically study young colonies grown for a small number of days and in which no morphological differentiation is typically visible. Our results indicate that several compounds form gradients, resulting in a metabolically heterogeneous population. Colonies are thus complex environments with numerous gradients creating micro-environments and metabolic heterogeneity, resulting in important physiological consequences for each subpopulation and for the colony as a collective^64,65^.

Our study adds a type of heterogeneity to a growing body of work describing metabolic heterogeneity in yeast colonies and biofilms^66^. Importantly, the heterogeneity we detected within young colonies is different compared with that observed in ageing yeast colonies on rich media, where complex metabolic changes resulted in the differentiation into upper- and lower-layer cells with vastly different physiologies^65,67,68^. Whereas in ageing colonies fermentative metabolism and growth take place in outer cells while inner cells appear stressed and starved, we observed the opposite in young, growing colonies where the fermentative population is the one close to the agar. More recently, another line of evidence has described a type of heterogeneity in glucose-limited yeast colonies where cells differentiate into dark and light types after several days in glucose-poor but amino acid-rich media^69^. Here, one population produces trehalose from aspartate which is then used by a second, glycolytic population of cells^70^. Here again, the key difference is that prolonged starvation was used to induce marked morphological and metabolic differentiation.

An intriguing question to explore in future studies is the nature of the interaction between pre- and postdiauxic populations and its impact on the fitness of the whole community. Cells can transition between populations (due to the higher growth rate of the bottom population, there has to be a net flux of cells into the top population over time because both populations remain relatively equal in size). Simultaneous utilization of glucose and ethanol could affect the overall fitness of the colony by minimizing the amount of ethanol diffusing back into the medium—this could be beneficial or detrimental in the light of competition for carbon or the antimicrobial effect of ethanol on competitors. Higher ethanol concentrations in the top layers might also decrease the risk of invading bacterial species, allowing those cells close to the nutrient source to exploit it efficiently.

Finally, we found that metabolic differences between subpopulations can affect cellular responses, as demonstrated for the clinically applied antifungal amphotericin B. In a recent study^24^ we showed that colonies containing cells with varying degrees of metabolic interactions have increased drug tolerance. While this previous work shows that metabolic heterogeneity is a source of antifungal tolerance in a fungal system where metabolic heterogeneity is genetically driven, we now show that heterogeneity in prototrophic wild-type colonies can also induce a differential response to antifungal drugs at the subpopulation level. This finding could be of critical importance in regard to yeast pathogens such as *Candida albicans* that trigger drug-tolerant infections by the formation of biofilms. In spatially structured communities it can be hard to disentangle the effect of physical shielding from metabolic heterogeneity but, like others^71^, we have found that heterogeneous resistance is maintained even when the spatial structure is disrupted. On the other hand, even spatially homogeneous (but stationary—that is, starved) liquid cultures can display some degree of heterogeneous resistance^72^. Hence, understanding the influence of metabolic heterogeneity on phenotypic diversity in these systems could have broad implications for future treatment development.

## Methods

### Yeast strains and media

A prototrophic derivative of the S288C-descendent standard laboratory strain BY4741 (*MAT*a *his3Δ1 leu2Δ0 met15Δ0 ura3Δ0*) was used throughout this study (described in (ref. ^24^)). It was obtained by repairing the four auxotrophies via integration of the missing genes into their native genomic loci. Cells were revived from cryostocks by plating out on yeast extract peptone dextrose agar (1% yeast extract, 2% peptone, 2% glucose and 2% agar) and incubated for 1–3 days. SM medium was prepared from yeast nitrogen base without amino acids (as 2× stock, stored in the dark; Sigma-Aldrich, no. Y0626) and ^13^C-labelled glucose (prepared as 10% stock; Sigma-Aldrich, no. 389374), to which l-lysine (prepared as 20 mM stock; Sigma-Aldrich, no. L5501) was added as required. Media components were sterilized by filtration. Where applicable, autoclaved agar was included in media at a final concentration of 2%. In SM medium, a glucose concentration of 1% was used throughout this study. Cultures and colonies were incubated at 30 °C. For experiments where cells were grown in/on ^13^C glucose medium, precultures/colonies were grown on SM medium containing ^13^C glucose without additional supplements to minimize carryover of ^12^C material.

### Physical separation of top and bottom cells in colonies

Hot agar medium was filled into 12-well tissue culture plates. Before it solidified, a small square of plastic (cut from a tip box lid) with a circular hole in the middle (made using a regular office hole punch) was dropped onto the agar surface. Colonies were then inoculated using a pipette tip and grown for three days. To harvest top cells, a plastic cell scraper was swiped over the plastic surface, applying even and gentle pressure. Cells were washed off the scraper and the remaining bottom layer of the colony was washed off the agar surface with water. The optical density (OD) of both suspensions was measured, followed by centrifugation (14,000*g*, 4 min) and removal of the supernatant. Proteomic samples were then prepared as described below.

### Measurement of free intracellular amino acids

Intracellular amino acids were extracted and measured as previously described^31,73^. In brief, 180 µl of 80% ethanol in water was added to previously frozen cell pellets. The sample was then incubated in a water bath at 80 °C for two min, followed by vigorous mixing for two min and a further two min at 80 °C. The extract was cleared by centrifugation (3,200*g*, five min) and used directly for LC–MS/MS analysis (Agilent 1290 Infinity HPLC, Agilent 6470 triple quadrupole mass spectrometer). Five microlitres of sample was separated by hydrophilic interaction chromatography on an analytical column (Waters ACQUITY UPLC BEH amide 2.1 × 100 mm^2^, 1.7 µm) maintained at 25 °C and a flow rate of 0.6 ml min^–1^. The starting conditions were 15% buffer A (1:1 acetonitrile/water, 10 mM ammonium formate, 0.176% formic acid) and 85% buffer B (95:5:5 acetonitrile/methanol/water, 10 mM ammonium formate and 0.176% formic acid). Starting conditions were maintained for three min followed by ramping to 5% buffer B over seven min, which was maintained for one min before returning to starting conditions. Total run time was 12.7 min. Source parameters were set as follows: gas temperature 325 °C, gas flow 10 l min^–1^, nebulizer 40 psi, sheath gas temperature 350 °C, sheath gas flow 11 l min^–1^, capillary voltage 3,500 V, nozzle voltage 1,000 V. Lysine was measured in positive mode by monitoring transitions 147–84 for unlabelled lysine and 153–89 for labelled lysine (fragmentor 80 and collission energy (CE) 10 for both). Data were analysed in MassHunter (Agilent). Correct peaks were identified by matching retention times to pure analytical amino acid standards, as well as a qualifier transition at 147.1–130.1 (fragmentor 80, CE 5). Labelled and unlabelled lysine were quantified by peak integration, both being reported as a fraction of total area (labelled + unlabelled).

### Proteomics sample preparation

For cells grown on agar media, colonies were washed off the surface with one ml of water, transferred to a 96-deep-well plate, pelleted by centrifugation (3,200*g*, four min) and frozen at −80 °C until further processing. For liquid cultures, roughly 1 OD-unit of cells were transferred to a 96-deep-well plate, separated from medium by centrifugation, resuspended in one ml of water, pelleted by centrifugation and frozen at −80 °C until further processing.

Samples were prepared by mechanical lysis in denaturing urea buffer, followed by reduction-alkylation of cysteine residues, digestion, solid phase extraction (SPE) and buffer exchange, as described previously^74,75^. In brief, 200 µl of lysis buffer (seven M urea and 0.1 M ammonium bicarbonate in water) and a small amount of acid-washed glass beads (425–600 µm in size) were added to each well and the plate sealed with a rubber seal mat. Cells were then lysed mechanically for 2 × 5 min using a 1600 MiniG bead mill (Spex Sample Prep) operated at 1,500 rpm. Then, 20 µl of 55 mM dithiothreitol was added with incubation at 30 °C for one h, followed by the addition of 20 µl of 120 mM iodoacetamide and a further 30 min incubation at room temperature in the dark. Next, one ml of 0.1 M ammonium bicarbonate was added to each well and the extract cleared by centrifugation (3,220*g*, five min). This was followed by transfer of 230–920 µl (varying between experiments, depending on the starting amount of biological material) to a fresh plate containing either 10–20 µl of trypsin solution (100 µg ml^–1^; Sequencing Grade Modified Trypsin, Bulk Sale Size, Promega, prepared according to the manufacturer’s instructions) or 10–20 µl of gluC solution (100 µg ml^–1^; New England Biolabs, prepared according to the manufacturer’s instructions). Proteins were digested at 37 °C overnight. Formic acid (prepared as a 10% stock) was added to a final concentration of 1% and peptides purified by SPE using 96-well SPE plates (BioPure Macro 96, PROTO 300 C18, no. HNS S18V-L, Nest Group). These were first conditioned with methanol, followed by 2× buffer B (50% acetonitrile in water) and 3× buffer A (3% acetonitrile in water with 0.1% formic acid). Samples were then loaded and washed three times with buffer A before elution into a fresh plate; 200 µl was used for all conditioning and wash steps, and elution was done using 2× 120 µl followed by 130 µl of buffer B. Samples were then dried at 45 °C in a Concentrator Plus (Eppendorf) using the V-AQ programme. Samples were reconstituted in 20–50 µl of buffer A, cleared of any insoluble components (3,200*g*, five min) and transferred to a fresh plate compatible with our autosampler. Peptide concentrations were estimated using absorption at 280 nm (Lunatic plate reader, Unchained Labs).

### Microflow LC–MS setup and measurements

Sample volumes containing two µg of peptides were analysed on a nanoAcquity UPLC (Waters) connected to a SCIEX TripleTOF 6600 with a DuoSpray Turbo V source, as described previously^75^. The column (Waters HSS T3, 150 mm × 300 µm, 1.8 µm particles) was maintained at 35 °C and a flow rate of five µl min^–1^. The chromatographic gradient was 20 min, starting with 3% buffer B and 97% buffer A and ending at 80% buffer B before returning to starting conditions (total run time, 27.5 min). Ion source gas 1 (nebulizer gas), ion source gas 2 (heater gas) and curtain gas were set to 15, 20 and 25, respectively. The ion spray voltage was set to 5,500 V and source temperature to 75 °C. The mass spectrometer was operated in high-resolution mode.

For DIA–sequential windowed acquisition of all theoretical fragment (DIA–SWATH) analysis (Supplementary Datasets 4 and 5) a SWATH method with 40 windows and 35 ms accumulation time was used, covering a precursor range of 400–1,250 *m/z*^75^. Data were analysed in DIA–NN v.1.8 (ref. ^32^) using a spectral library generated by gas phase fractionation and scanning SWATH analysis^74^ with long gradients on the same physical setup^75^. The library contained 4,936 protein groups and 58,599 precursors. Sciex wiff data files were loaded directly into DIA–NN. MS2 and MS1 mass accuracy was set to 20 and 12, respectively. ‘Use isotopologues’ and ‘Remove likely interferences’ were enabled, with ‘Robust LC (high precision)’ set for quantification strategy. Reverse-transcription-dependent cross-run normalization was enabled, and gene quantities as reported by DIA–NN (which internally uses maxLFQ^76^) were used for differential expression analysis.

For targeted measurements (PRM) (Supplementary Dataset 2), suitable peptides were selected based on their amino acid composition (exactly two lysine residues and no cysteine or methionine residues), the position of the lysine residues in the peptide (ideally one lysine close to the C terminus and both lysines far apart from each other), their typical abundance, as well as other quality indicators (consistency of identification across runs, no probable interferences reported and consistency of retention time across runs). Only proteotypic peptides were used. PRM methods were generated using Skyline^77^. Generally, all amino acids except lysine were set to carry structural modifications to reflect their ^12^C-to-^13^C mass shift, and isotope label modification with matching retention time was applied to lysine. Isotope labels were then permuted fully, resulting in four uniquely labelled precursors per peptide of interest. Collision energies and declustering potentials were predicted using the SCIEX setting. For Supplementary Dataset 2, samples were run on the setup described above with identical chromatography and source parameters. For data underlying Extended Data Fig. 1, samples were run on high-flow chromatography (Agilent 1290 Infinity II) with a five min gradient on a Infinitylab Poroshell 120 EC-C18 column (2.1 × 50 mm^2^, 1.9 µm) coupled to a SCIEX TripleTOF 6600 with IonDrive source.

Data files were directly loaded into Skyline v.21.1 or v.21.2. Skyline was instructed to extract data for single-charged y and b fragment ions, starting with ion3 and ending with last ion-2, as well as for intact precursors. Resolving powers were set to 20,000 for MS1 and 12,000 for MS2, with high-selectivity extraction enabled. Identification and integration were checked manually and quantification reports generated for further analysis in python. For fragment-level quantification, only fragments containing exactly one lysine were used. This allowed us to resolve and quantify the two mixed-labelling states (which have the exact same precursor mass), because fragments with one lysine are unique to either one of the two. Only the top three abundant fragments (average rank across all samples) were used for quantification. For each fragment the ratios of the different labelling states were determined first before averaging across fragments, peptides and replicates.

### Observed and expected labelling state abundance

Lysine incorporation into peptides was modelled as a Bernoulli process with two trials (one per lysine residue). Probability was derived from the abundances of the three labelling states by computing the average occurrence of imported lysine across the two sites (*P* = (0 x producer peptide abundance + 1 x mixed peptide abundance + 2 x consumer peptide abundance)/2). The expected relative abundance, *r*, of labelling states was then computed as

$$r = \left( {\begin{array}{*{20}{c}} 2 \\ k \end{array}} \right)P^k(1 - P)^{2 - k}$$

where *k* is the number of imported lysine residues in the peptide.

### Nanoflow LC–MS setup for DIA-PASEF measurements

For proteome-wide determination of producer and consumer gene expression differences (Supplementary Dataset 3), tryptic digests were prepared from colonies and liquid cultures as described above. Peptides (400 ng) were analysed on a nano-flow chromatography setup (UltiMate 3000, Thermo Scientific Dionex) coupled to a TIMS quadrupole time-of-flight instrument (timsTOF Pro2, Bruker Daltonics). We used a 25 cm Aurora Series analytical column with emitter column (CSI, 25 cm × 75 µm ID, 1.6 µm C18, IonOpticks) maintained at 50 °C. Mobile phases A and B (water with 0.1% formic acid and acetonitrile with 0.1% formic acid, respectively) were applied on a linear gradient starting from 2% B and increasing to 17% by minute 87, followed by an increase to 25% B to minute 93, 37% B to minute 98 and 80% B to minute 99, which was maintained until minute 104. The column was then equilibrated in 2% B for the next 15 min. For calibration of the ion mobility dimension, three of the Agilent ESI-Low Tuning Mix ions were selected (*m/z* (Thomson (Th)), 1/K_0_ (Vs cm^-2^): 622.0289, 0.9848; 922.0097, 1.1895; 1221.9906, 1.3820). Data were acquired in DIA–PASEF mode. In the *m/z* dimension, windows ranged from 400 to 1,200 Th and in the 1/K_0_ dimension from 0.6 to 1.4 Vs cm^−2^, with 32 × 25 Th windows. Collision energy was decreased linearly, from 59 eV at 1/K_0_ = 1.3 Vs cm^−2^ to 20 eV at 1/K_0_ = 0.85 Vs cm^−2^.

For the spike-in experiment shown in Extended Data Fig. 4e–g, 500 ng of total peptides (pooled sample from the experiment shown in Fig. 3 and Supplementary Dataset 3 plus fully labelled ^13^C peptides) was analysed using Evosep chromatography (EVOTIP PERFORMANCE, set up according to the manufacturer’s protocol), with the EVOSEP 15 SPD LC method (88 min gradient) and the EV1137 PERFORMANCE column (15 cm × 150 µm, 1.5 µm at 40 °C), coupled to a 10 µm Zero Dead Volume Captive Spray Emitter (Bruker, no. 1865691). The same mass spectrometer and acquisition method were used.

Data were analysed using the recently developed tims module in DIA–NN 1.8 (ref. ^34^). In a first step, the spectral library described above was modified in silico to reflect the ^13^C-labelling state of colonies grown on ^13^C glucose with ^12^C lysine. For this, fixed modifications of the type ‘label’ (indicating that they do not affect retention time) were applied to all amino acids except for lysine, where the same was applied as a variable modification. Only precursors with charge +2 or +3 and of length 7–30 residues were included in the modified library. The ‘ExcludeFromAssay’ column of the library was then set to True for all b-series ions and False for all y-series ions, indicating that only y-series ions (containing exactly one lysine) should be used for quantification, thereby excluding fragments not unique to one of the labelling states of the precursors. Raw data files were then directly loaded into DIA–NN and analysed with the previously generated library. MS1 and MS2 mass accuracies were set to 10, ‘Use isotopologues’ was disabled, ‘Remove likely interferences’ was enabled and ‘Robust LC (high precision)’ was set for Quantification Strategy; ‘–restrict-fr’ was added to the option field to enable the use of the ‘ExcludeFromAssay’ column of the library. Default options were used otherwise.

The DIA–NN output reports were further processed in python. Only proteotypic peptides with exactly one lysine residue located at the end of the peptide and with Quantity.Quality >0.7 were included in the analysis. For the experiment investigating colony subpopulations, two out of eight samples were excluded from the analysis (one had a low number of IDs and one a median ratio of labelled/unlabelled peptides that differed substantially from the other seven). Matching labelled/unlabelled precursor pairs were identified based on Data.File, Stripped.Sequence and Precursor.Charge, and the ratio of the Precursor.Quantity of heavy (producer) to light (consumer) was computed. For each sample separately, heavy/light rations were divided by the sample median and log_2_ transformed. It was then tested whether the mean ratio across replicates was significantly different from 0, using the 'ttest_1samp' function from scipy.stats^78^. *P* values were corrected for multiple testing using the method of Benjamini and Hochberg. Precursors were considered significant if the adjusted *P* value was <0.05. Median heavy light ratio, as well as other summary statistics, were generated. Precursor-level results were then aggregated at the gene level, considering only precursors identified in at least three samples. A gene was considered a hit if at least one precursor was significant, if the absolute average log_2_-transformed median FC across all precursors was >0.75 and if all precursors showed the same trend (all median log_2_(FC) have the same sign).

Gene enrichment analyses were performed with gProfiler^79^, accessed via python API (gprofiler-official v.1.0.0). Gene Ontology enrichments were visualized with the CellPlot package (S. E. Templer and R. Sehlke). Yeast pathways were downloaded manually from pathway.yeastgenome.org (accessed 8 August 2021). Genes annotated to specific Gene Ontolgy terms (oxidative phosphorylation: GO:0006119; cytoplasmic translation: GO:0002181) were retrieved with gProfiler on 9 August 2021. The pathway map was drawn with iPATH^80^. For this, Saccharomyces Genome Database gene names were first converted to UNIPROTSWISSPROT IDs using gProfiler. Edges were coloured according to the direction of change and drawn with a thickness reflecting log_2_-transformed FC (width, 1 + log_2_(FC) × 10).

Quantitative changes in gene expression between postdiauxic growth compared with early exponential growth (Figs. 3f and 4b) were obtained from Supplementary Data 1 of (ref. ^38^). This dataset contains 10-plex tandem mass tag measurements of yeast strain DBY7286 (*MATa, ura3, GAL2*) at ten time points during growth on liquid yeast extract peptone dextrose medium. We used protein-level mean values from the sheet ‘timecourse statistics’ and divided the 33 h timepoint (timepoint 10, late postdiauxic growth and entry into stationary phase) by the 11 h time point (time point 3, early exponential growth), followed by log_2_ transformation.

### FACS of amphotericin-treated cells

Heterogeneous and ^13^C-labelled colonies were grown on SM medium with 1% ^13^C glucose and 200 µM lysine for three days. Colonies were then resuspended in 1.1 ml of SM with 0.2% ^13^C glucose, and 500 µl was added to a similar volume of amphotericin B solution followed by mixing and incubation for one h as described above. Cells were collected by centrifugation and resuspended in PBS. Before FACS, cells were sonicated for 20 s at 50 W (JSP Ultrasonic Cleaner model US21) to increase singlet efficiency. Cells were then stained with 8 µg ml^–1^ propidium iodine to identify live and dead cells, before FACS analysis. Live and dead cells were sorted on a BD Aria Fusion with BD FACSDiva (v.8.0.1) software (BD Biosciences) using a 488 nm excitation laser. The gating strategy is illustrated in Extended Data Fig. 7a. Sorted cells were collected by filtration through a 0.45 µm polyvinylidene difluoride membrane (Agilent, no. 200959–100) and washed from the filter with 200 µl of proteomics lysis buffer, followed by sample processing and targeted PRM measurement as described above.

### Assessment of amphotericin B resistance by flow cytometry

To assess the effect of lysine on amphotericin resistance in colonies, colonies were grown for two days with or without 400 µM lysine in SM medium with 1% standard (^12^C) glucose. Colonies were resuspended in one ml of SM with 0.2% ^12^C glucose and OD_600_ was determined. To assess differential resistance in the top and bottom cell layers in colonies, non-supplemented colonies were grown in SM medium with 1% ^12^C glucose for three days. Top cells were scraped off as described above and resuspended in 550 µl of SM with 0.2% ^12^C glucose, and bottom cells were washed from the agar with the same volume. The next step in both experiments was the addition of 200 µl of cell suspension to 200 µl of amphotericin B solution, followed by shaking at 1,000 rpm for five min and incubation at room temperature in the dark. Cell death was assessed using the LIVE/DEAD Fixable Far Red Dead Cell Stain Kit for 633 or 635 nm excitation (ThermoFisher Scientific, no. L10120) according to the manufacturer’s instructions. Cells were then sonicated for 20 s at 50 W (JSP Ultrasonic Cleaner model US21) to increase singlet efficiency, and 250 µl was transferred to a 96-well plate for analysis. A total of 20,000–30,000 cells per sample were measured in a Fortessa X20 Flow cytometer (BD Biosciences) using the HTS plate mode on BD Diva software, v.8.0.1 and a 633 nm excitation laser, to capture dye fluorescence intensity. Populations of interest were gated using FlowJo v.10.3.0, as illustrated in Extended Data Figs. 8 and 9.

### Reporting summary

Further information on research design is available in the Nature Portfolio Reporting Summary linked to this article.

### Supplementary information

Supplementary Information Supplementary Note 1.

Reporting Summary

Supplementary Data. Supplementary Datasets 1–7.

## Figures and Tables

**Extended Data Table. 1:**
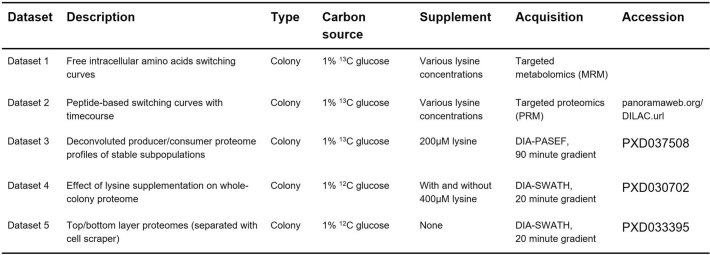
Overview of mass spectrometry datasets generated in this study

## Data Availability

Nine Extended Data figures and one Extended Data table are provided with this manuscript. Seven Supplementary datasets are supplied in xlsx format. For DIA experiments: raw data, DIA–NN pipelines, log and report files, as well as code used for analysis, have been deposited at ProteomeXchange^81^ via PRIDE^82^, with the following accessions: PXD037508 (Supplementary Dataset 3 and spike experiment shown in Extended Data Fig. 4e–g); PXD030702 (Supplementary Dataset 4); PXD033395 (Supplementary Dataset 5). For targeted proteomics experiments, Skyline files, raw data and Jupyter notebooks containing code used for analysis and plotting have been deposited with Panorama Public^83^ and ProteomeXchange: https://panoramaweb.org/DILAC.url (10.6069/s9b3-zz35) and PXD036959.
