# Supplementary material for: Metabolic heterogeneity and cross-feeding within isogenic yeast populations captured by DILAC
Source: Nat Microbiol. 2023 Feb 16;8(3):441–54. doi: 10.1038/s41564-022-01304-8 (PMC9981460; doi:10.1038/s41564-022-01304-8)
Supplement: Supplementary file 1 — Supplementary Note 1. [file 41564_2022_1304_MOESM1_ESM.pdf]

nature microbiology

Article

https://doi.org/10.1038/s41564-022-01304-8

Metabolic heterogeneity and cross-feeding within isogenic yeast populations captured by DILAC

In the format provided by the authors and unedited

Supplementary Note 1

Additionally to the proteins described in the main text, we note that aconitase Aco2p is up- rather than downregulated in lysine producers (**Dataset 3:** fc=1.63, 3/3 peptides significant) (Fig. 3F). This particular deviation can be attributed to lysine availability as we see concordant gene expression changes in colonies supplemented with lysine. In the presence of lysine, Aco2p is downregulated (**Dataset 4:** fc=0.47, p_{adj}=0.003), presumably due to its direct role in lysine biosynthesis[1]. Furthermore, Pdc6p was strongly upregulated in producers (**Dataset 3**: fc=6.6, 2/2 peptides significant). Despite not being differentially expressed in the specific experiment by ref[2], this minor pyruvate decarboxylase isozyme is known to be induced during growth on ethanol[3] and serves an additional role in amino acid catabolism[4]. It is also induced by sulphur starvation[5] which could indicate a sulphur limitation of top cells, although the observed effect could be due to carbon source and/or amino acid catabolism alone.

1. Fazius, F., Shelest, E., Gebhardt, P. & Brock, M. The fungal α-aminoadipate pathway for lysine biosynthesis requires two enzymes of the aconitase family for the isomerization of homocitrate to homoisocitrate. *Mol. Microbiol.* **86**, 1508–1530 (2012).
2. Murphy, J. P., Stepanova, E., Everley, R. A., Paulo, J. A. & Gygi, S. P. Comprehensive Temporal Protein Dynamics during the Diauxic Shift in Saccharomyces cerevisiae. *Mol. Cell. Proteomics* **14**, 2454–2465 (2015).
3. Hohmann, S. Characterization of PDC6, a third structural gene for pyruvate decarboxylase in Saccharomyces cerevisiae. *J. Bacteriol.* **173**, 7963–7969 (1991).
4. Dickinson, J. R., Salgado, L. E. J. & Hewlins, M. J. E. The catabolism of amino acids to long chain and complex alcohols in Saccharomyces cerevisiae. *J. Biol. Chem.* **278**, 8028–8034 (2003).
5. Boer, V. M., de Winde, J. H., Pronk, J. T. & Piper, M. D. W. The genome-wide transcriptional responses of Saccharomyces cerevisiae grown on glucose in aerobic chemostat cultures limited for carbon, nitrogen, phosphorus, or sulfur. *J. Biol. Chem.* **278**, 3265–3274 (2003).
