# Supplementary material for: Metabolic heterogeneity and cross-feeding within isogenic yeast populations captured by DILAC
Source: Nat Microbiol. 2023 Feb 16;8(3):441–54. doi: 10.1038/s41564-022-01304-8 (PMC9981460; doi:10.1038/s41564-022-01304-8)
Supplement: Supplementary file 2 — Reporting Summary [file 41564_2022_1304_MOESM2_ESM.pdf]

nature portfolio

Corresponding author(s): Markus Ralser

Last updated by author(s): Dec 6, 2022

Reporting Summary

Nature Portfolio wishes to improve the reproducibility of the work that we publish. This form provides structure for consistency and transparency in reporting. For further information on Nature Portfolio policies, see our Editorial Policies and the Editorial Policy Checklist.

Statistics

For all statistical analyses, confirm that the following items are present in the figure legend, table legend, main text, or Methods section.

n/a	Confirmed	
☐	☒	The exact sample size (n) for each experimental group/condition, given as a discrete number and unit of measurement
☐	☒	A statement on whether measurements were taken from distinct samples or whether the same sample was measured repeatedly
☐	☒	The statistical test(s) used AND whether they are one- or two-sided<br>*Only common tests should be described solely by name; describe more complex techniques in the Methods section.*
☒	☐	A description of all covariates tested
☐	☒	A description of any assumptions or corrections, such as tests of normality and adjustment for multiple comparisons
☐	☒	A full description of the statistical parameters including central tendency (e.g. means) or other basic estimates (e.g. regression coefficient) AND variation (e.g. standard deviation) or associated estimates of uncertainty (e.g. confidence intervals)
☐	☒	For null hypothesis testing, the test statistic (e.g. F, t, r) with confidence intervals, effect sizes, degrees of freedom and P value noted<br>*Give P values as exact values whenever suitable.*
☒	☐	For Bayesian analysis, information on the choice of priors and Markov chain Monte Carlo settings
☒	☐	For hierarchical and complex designs, identification of the appropriate level for tests and full reporting of outcomes
☐	☒	Estimates of effect sizes (e.g. Cohen's d, Pearson's r), indicating how they were calculated

Our web collection on statistics for biologists contains articles on many of the points above.

Software and code

Policy information about availability of computer code

Data collection

Proteomics data acquisition - Sciex Analyst Instrument Control Software v1.7.1 for TripleTOF 6600
Proteomics data acquisition - Bruker Instrument Control Software for timsTOF Pro2 v1.1.19 68
Metabolomics data acquisition - Agilent MassHunter Instrument Control Software for QQQ 6470 v8.07.000
Flow cytometry data acquisition - DIVA Instrument Control Software v8.0.1 (BD Instruments)

Data analysis

Proteomics data analysis - DIA-NN v 1.8
Metabolomics data analysis - Agilent MassHunter Quantitative Analysis for QQQ v8.07.00
General data analysis - Python v3.7
Flow Cytometry - FlowJo v10.3.0
GO enrichment - gProfiler (gprofiler-official v1.0.0)
Gene expression visualisation - iPATH v3.0
GO enrichment visualisation - CellPlot v1.0

For manuscripts utilizing custom algorithms or software that are central to the research but not yet described in published literature, software must be made available to editors and reviewers. We strongly encourage code deposition in a community repository (e.g. GitHub). See the Nature Portfolio guidelines for submitting code & software for further information.

Data

Policy information about availability of data

All manuscripts must include a data availability statement. This statement should provide the following information, where applicable:

- Accession codes, unique identifiers, or web links for publicly available datasets
- A description of any restrictions on data availability
- For clinical datasets or third party data, please ensure that the statement adheres to our policy

9 Extended Data Figures and 1 Extended Data Table are provided with this manuscript.

7 Supplementary Datasets are supplied in xlsx format.

For DIA experiments: Raw data, DIA-NN pipelines, log and report files, as well as code used for analysis have been deposited to ProteomeXchange via PRIDE with the following accessions:
- PXD037508 (Dataset 3 and spike experiment shown in Extended Data Fig. E-G)
- PXD030702 (Dataset 4)
- PXD033395 (Dataset 5)
For targeted proteomics experiments: Skyline files, raw data and Jupyter notebooks containing code used for analysis and plotting have been deposited to Panorama Public and ProteomeXchange: panoramaweb.org/DILAC.url (doi:10.6069/s9b3-zz35) and PXD036959.

This manuscript relied on the following public databases and datasets:
- Quantitative changes in gene expression between post-diauxic growth compared to early exponential growth (Fig. 3E+F and Extended Data Fig. 64B) - Taken from Supplementary Data 1 of Murphy et al. (2015) "Comprehensive Temporal Protein Dynamics during the Diauxic Shift in Saccharomyces cerevisiae" Mol. Cell. Proteomics. DOI: 10.1074/mcp.M114.045849
- Yeast reference proteome UP000002311 - obtained from uniprot.org
- Yeast pathway database - pathway.yeastgenome.org (accessed 8.9.2021)
- GO annotation and gene ID database - gProfiler (biit.cs.ut.ee/gprofiler, accessed 8.9.2021)

Human research participants

Policy information about studies involving human research participants and Sex and Gender in Research.

Reporting on sex and gender	NA
Population characteristics	NA
Recruitment	NA
Ethics oversight	NA

Note that full information on the approval of the study protocol must also be provided in the manuscript.

Field-specific reporting

Please select the one below that is the best fit for your research. If you are not sure, read the appropriate sections before making your selection.

☒ Life sciences ☐ Behavioural & social sciences ☐ Ecological, evolutionary & environmental sciences

For a reference copy of the document with all sections, see nature.com/documents/nr-reporting-summary-flat.pdf

Life sciences study design

All studies must disclose on these points even when the disclosure is negative.

Sample size	We determined required sample sizes by statistical power calculations. For differential gene expression testing (Dataset 3), with a typical CV of 10% in proteomics experiments, 6 replicates are sufficient to detect a 2-fold difference in expression with a power of >99%, even after Bonferroni correction for the number of genes in the yeast proteome (approx 6000).
Data exclusions	For the experiment investigating colony sub-populations (Dataset 3, Figure 3), 2 out of 8 samples were excluded from the analysis (one had a low number of IDs and one a median ratio of labelled to unlabelled peptides which differed substantially from the other 7). This is stated in the Methods section. Exclusion criteria were not pre-established but exclusion of (this small number) of samples was done before any down-stream analysis such as differential gene expression testing.
Replication	Statistical analyses and conclusions are supported by sufficient replicates, both within each experiment, as well as through independent

Replication	experiments (performed on different days). The key finding of Figure 2 (that lysine producer/consumer subpopulations exist in colonies) is supported by an independent experiment, as well as orthogonal data analysis approaches (shown in Supplementary Figure 2). The key findings of Figure 3 (diauxie-like heterogeneity) and Figure 4 (metabolic heterogeneity confers differential resistance to amphotericin B) are supported by independent and orthogonal experiments further exploring underlying mechanisms.
Randomization	No group allocation took place.
Blinding	No group allocation took place.

Reporting for specific materials, systems and methods

We require information from authors about some types of materials, experimental systems and methods used in many studies. Here, indicate whether each material, system or method listed is relevant to your study. If you are not sure if a list item applies to your research, read the appropriate section before selecting a response.

Materials & experimental systems

n/a	Involved in the study	
☒	☐	Antibodies
☒	☐	Eukaryotic cell lines
☒	☐	Palaeontology and archaeology
☒	☐	Animals and other organisms
☒	☐	Clinical data
☒	☐	Dual use research of concern

Methods

n/a	Involved in the study	
☒	☐	ChIP-seq
☐	☒	Flow cytometry
☒	☐	MRI-based neuroimaging

Flow Cytometry

Plots

Confirm that:

☒ The axis labels state the marker and fluorochrome used (e.g. CD4-FITC).

☒ The axis scales are clearly visible. Include numbers along axes only for bottom left plot of group (a 'group' is an analysis of identical markers).

☒ All plots are contour plots with outliers or pseudocolor plots.

☒ A numerical value for number of cells or percentage (with statistics) is provided.

Methodology

Sample preparation	Flow Cytometry<br>Cell death was assessed using the LIVE/DEAD™ Fixable Far Red Dead Cell Stain Kit, for 633 or 635 nm excitation (ThermoFisher Scientific, Cat no. L10120) according to the manufacturer's instructions. Cells were then sonicated for 20 s at 50W (JSP Ultrasonic Cleaner model US21) to increase singlets efficiency, and 250 µL were transferred to a 96-well plate for HPT-FC analysis.<br><br>FACS<br>Cells were collected by centrifugation and resuspended in PBS. Prior to FACS, cells were sonicated for 20 s at 50W (JSP Ultrasonic Cleaner model US21) to increase singlets efficiency. Cells were then stained with 8 µg/mL propidium iodine, to identify live and dead cells, prior to FACS.
Instrument	Flow Cytometry<br>Fortessa X20 Flow cytometer (BD Biosciences)<br><br>FACS<br>Aria Fusion (BD Biosciences)
Software	BD FACSDiva (v8.0.1) for instrument control and sorting<br>FlowJo v10.3.0
Cell population abundance	FOR FACS, at least 4 million cells were present in each sorted population. As sorted populations were immediately processed for proteomics and conservative gating was used, no post-sorting purity check was required.
Gating strategy	FSC-A vs SSC-A was used to identify singlet cells. Gating for cell viability was done based on live (fresh yeast cells) and dead (cells incubated at 90°C for 10 minutes) as well as unstained control samples.

☒ Tick this box to confirm that a figure exemplifying the gating strategy is provided in the Supplementary Information.
